# Reply to: Videomicroscopic investigation of the microcirculation requires uniform definitions

**DOI:** 10.14814/phy2.13304

**Published:** 2017-06-11

**Authors:** Ian Wright

**Affiliations:** ^1^ Illawarra Health and Medical Research InstituteUniversity of WollongongNew South WalesAustralia

We welcome Erdem et al.'s insightful comments on our largest reported series of video microcirculatory values for the term newborn.

Firstly, as Erdem et al. correctly point out, in editing we have conflated the pre‐existing imaging version using OPS and the subsequent SDF technology, which they correctly identify as the one used, as described in Figure 2.1 of his recent excellent review (Milstein et al. 2012). We recommend this to understand the differences between the technologies.

We believe that the other differences that have been commented on are entirely the difference between results from a population, rather than from an individual. As the number of values may vary for different assessments (the large vessels being the most obvious example) the means or medians can indeed present apparent anomalies. In order to provide the fullest dataset we have not restricted the values to those samples where only a full set in all images in all criteria are complete, as this may bias the applicability of the final observations to the whole term newborn population.
